# Autonomy dimensions and care seeking for delivery in Zambia; the prevailing importance of cluster-level measurement

**DOI:** 10.1038/srep22578

**Published:** 2016-03-02

**Authors:** Sabine Gabrysch, Shannon A. McMahon, Katja Siling, Michael G. Kenward, Oona M. R. Campbell

**Affiliations:** 1 Ruprecht-Karls-Universität Heidelberg, Institute of Public Health; 2 London School of Hygiene & Tropical Medicine, Faculty of Epidemiology and Population Health.

## Abstract

It is widely held that decisions whether or when to attend health facilities for childbirth are not only influenced by risk awareness and household wealth, but also by factors such as autonomy or a woman’s ability to act upon her own preferences. How autonomy should be constructed and measured – namely, as an individual or cluster-level variable – has been less examined. We drew on household survey data from Zambia to study the effect of several autonomy dimensions (financial, relationship, freedom of movement, health care seeking and violence) on place of delivery for 3200 births across 203 rural clusters (villages). In multilevel logistic regression, two autonomy dimensions (relationship and health care seeking) were strongly associated with facility delivery when measured at the cluster level (OR 1.27 and 1.57, respectively), though not at the individual level. This suggests that power relations and gender norms at the community level may override an individual woman’s autonomy, and cluster-level measurement may prove critical to understanding the interplay between autonomy and care seeking in this and similar contexts.

Investigations into gender, power and decision-making have existed for decades. Early studies examined power relations and decision-making in American households in the 1960s 1. In the 1980s, literature on the relationship between maternal autonomy and health indicators in low-income settings emerged^2^. Women’s autonomy has been defined as women possessing “control over their own lives”^3^, being able to make decisions and act upon them^4^ and “to manipulate (their) personal environment”^2^.

Studies on the relationship between autonomy and population health proliferated following the 1994 Cairo International Conference on Population and Development, which declared that human advancement is inextricably linked to advances in gender equality and equity, to the empowerment of women via their ability to control decisions related to their reproductive health, and to the elimination of violence against women^5^,^6^,^7^,^8^. This global priority was reiterated in the Millennium Development Goals^9^, of which the third aims to promote gender equality and empower women, and the fifth aims to reduce maternal mortality by promoting delivery in the presence of a skilled birth attendant (which is synonymous with facility-based delivery across most low-income settings).

The implicit theory of maternal autonomy states that more autonomous women are better equipped to act upon their preferences related to reproductive health care - even when confronted with contradictory or competing preferences among others in their household (such as husbands, co-wives or mothers-in-law), or their broader community. To that end, increases in individual women’s autonomy have been positively associated with improvements in: care seeking during the antenatal period^10^; contraceptive use^11^ and birth spacing^12^; and child health^13^. Despite these correlations, increases in autonomy do not consistently lead to improvements in health indicators^14^.

Literature on autonomy and reproductive health has been described as “asymmetrical”^14^ with most studies conducted in Asia (particularly in South Asia) and relatively few undertaken in sub-Saharan Africa. Nevertheless, at least three studies have investigated how dimensions of autonomy affect care seeking for delivery in sub-Saharan Africa^15^.

A cross-sectional study in the slums of Nairobi, Kenya, found that women’s education and household wealth were strong predictors of facility delivery, but overall autonomy and the dimensions of decision-making and freedom of movement were not associated, or associated in a “counter-intuitive” direction. The authors report wealth modified the association, in that among the poorest women, those with lowest autonomy were most likely to deliver in a facility while the opposite held for the richest^14^.

Another cross-sectional study, drawing on Demographic and Health Survey (DHS) data from Ethiopia and Eritrea, found that most autonomy indices were not significantly associated with facility-based birth after controlling for confounders^16^. However, some autonomy dimensions did predict skilled birth attendance, namely maternal autonomy related to major purchases (in Eritrea only), and autonomy related to intimate partner violence (in Ethiopia only)^16^.

In a pooled analysis across 33 settings (including 21 African settings), Ahmed and colleagues^17^ found that women’s empowerment was associated with higher maternal health service utilization, and women with the highest decision-making power were 1.3 times more likely to attend facilities for birth compared to women with no decision-making power^17^. However, they also noted that of the three socio-economic factors examined (economic, educational and empowerment-related), female empowerment “was the least strong factor associated with women’s use of maternal health services in all (31) countries, and especially so among African countries”^17^.

While stakeholders continue to advocate research and interventions that consider the role of maternal autonomy^6^, the construct remains challenging to define, quantify, analyse and operationalize. In some settings, finding a word that adequately conveys the cognitive and semantic meaning of autonomy is difficult, and can vary widely – even across members of the same household^4^. In other cases, researchers highlight the complexity of measuring autonomy’s dimensions. For example, women may be able to decide routine purchases, but such decision-making may not extend to health care or family planning. Even within the same domain, women’s autonomy may vary, as described in a Kenyan study which found women had more autonomy related to treatment for childhood fever than for convulsions^18^.

Prominent researchers have also posited fundamental concerns about how autonomy is conceptualized, arguing that the term employed today represents Western, feminist values^7^, which stress the importance of the self as an independent, assertive, self-contained entity^19^. This independent or individualistic construal runs counter to an interdependent self, which is exemplified in several Asian and African settings and which emphasizes group harmony and social connectedness^19^. In interdependent settings, where attitudes, preferences and behaviours are “shaped and governed by a consideration of the reaction of others”^19^, it may be inappropriate or problematic to apply a Western notion of autonomy to study behaviour^7^. These authors advocate expanding studies to account for concepts of personhood defined “not as an isolated atomic individual, but one embedded in a web of social relationships”^7^ and that researchers consider how broader hierarchies create and reinforce inequities that are experienced across levels (individual, interpersonal, community) and ultimately shape the public health landscape^7^.

Using national data from Zambia, our study aims to incorporate this interdependent construal of self into an investigation of how female autonomy influences facility use for childbirth. As with previous studies, we studied several autonomy dimensions separately. We sought to build on existing understandings by examining the effect of autonomy at the individual and community level separately.

## Methods

This study uses data from the Zambia Demographic and Health Survey (DHS) in 2007 on 3200 births to 2012 married women (aged 15–49) living in 203 rural clusters. The DHS is a nationally representative survey and its primary sampling units or clusters usually correspond to villages in rural areas. Demographic and Health Surveys now incorporate explicit autonomy questions, wherein respondents are asked who has the final say on certain decisions in the household, as well as attitudes towards wife-beating, the right to refuse sex and the experience of violence. Also included is information on proxy indicators for autonomy such as age difference between spouses, age at first marriage and age at first birth^20^,^21^.

We used these variables to construct raw autonomy scores with five dimensions: financial, movement, health care, relationship and violence. For financial autonomy we used information about women’s decision-making power on large household purchases and control of own earnings. Freedom of movement was defined as a woman’s decision-making power concerning visits to her family and friends. The health care autonomy dimension was constructed using the specific question about decision-making on the woman’s own health care and whether getting permission is an obstacle to getting medical help for herself when she is sick. The woman’s position in the relationship was constructed using information on age at marriage and first birth, difference in age between spouses, polygamy, negotiation between the spouses and the woman’s attitude towards wife-beating and the right to refuse sex. The violence dimension used information on husband’s control behaviours, as well as of emotional, physical and sexual violence by the husband.

The component variables each contributed to the overall raw score of their dimension, whereby the answers reflecting a higher degree of autonomy added more points. For instance, late first marriage and a stated opposition to wife-beating or forced sex would yield higher autonomy scores. Details on the scoring system are provided in Appendix A (Table A.1) and a distribution of raw scores is shown in Appendix B (Table B.1). We used simple weights instead of more complex data reduction methods as this is more transparent.

We constructed community-level aggregate variables for the five autonomy dimensions to explore potential contextual effects. Raw scores of all interviewed women in the 203 clusters were averaged to construct these, thus also considering women who had not given birth in the last five years. We then constructed individual deviances from cluster averages by subtracting the raw scores from the cluster average scores. For instance, a woman living in a cluster with an average relationship autonomy score of 20 would be assigned an individual deviance of ‘−5’ if her raw score was 15, and a deviance of ‘+3’ if her raw score was 23. In this way we separated a cluster-level autonomy variable from an individual-level autonomy variable relative to the individual woman’s cluster. The distribution of the resulting scores for cluster-level averages and individual-level deviances is shown in Appendix B (Table B.2).

Women’s autonomy is likely to be associated with education, wealth, distance and other variables that can influence facility delivery. For this reason, we considered a wide range of potential individual-level confounders, including age, parity, multiple pregnancy, mode of delivery, previous C-section, literacy, education, occupation, ethnic group, religion, household composition, household wealth, health insurance, husband’s education, husband’s occupation, exposure to media, exposure to health information, fertility attitudes, as well as cluster-level confounders including average exposure to media, health information and average fertility attitudes in men and women, and average men’s support for skilled attendance at birth. Straight-line distance to delivery facilities and facilities’ level of Emergency Obstetric Care provision were also considered as confounders. These were calculated using information from the Zambia Health Facility Census 2005 and linked to the DHS dataset using GIS coordinates, as described earlier^22^.

We built separate models for each of the five autonomy dimensions, using three-level random effects logistic regression to account for the dependency between births to the same mother and in the same cluster in terms of facility delivery. The model was implemented in Stata 12 using the “xtmelogit” command. We studied the influence of the autonomy dimensions on facility delivery as a linear effect. The autonomy variables were parametrized as cluster-level averages (xav_i_) and individual deviances (xdev_ij_) from cluster averages to clearly separate cluster-level from individual-level effects. Both were used as separate predictors in the models relating them to the outcome, and could thus have different coefficients. The between-cluster coefficient (ß_B_) represents the influence of the cluster-level autonomy variable and the within-cluster coefficient (ß_w_) represents the influence of the individual woman’s autonomy deviance, i.e. her autonomy relative to her cluster mean. The model can be written as follows, for a birth k to a mother j in a cluster i: logit (Y_ijk_) = α + ß_B_* xav_i_ + ß_W_ * xdev_ij_ + u_i_ + s_ij_ + e_ijk_, with α being the intercept, u_i_ the cluster random effect, s_ij_ the mother random effect and e_ijk_ the error term.

We first examined the influence of each potential confounder separately on the relationship between autonomy and facility delivery. Those variables changing the log odds ratio of the individual deviance and/or cluster average autonomy by at least 10% were considered for inclusion in the final model. This was built by adding the potential confounders in the order of the strength of their confounding effects and retaining them if their inclusion still altered the log odds ratio of one or both autonomy variables by at least 10%.

To provide one interpretation of the associations found, we also computed predicted probabilities of facility delivery from the logistic regression models using so-called marginal standardization. This was done with the “margins” and “marginsplot” commands in Stata (specifying the option “predict (mu fixedonly)” to set the random effects term to 0). It predicts probabilities for every observed confounder value and then combines these as a weighted average for each exposure level^23^.

Information on place of delivery was missing for 10 births, and financial, movement and health care autonomy questions had less than 10 missing values each. Information on violence experience was not available for 333 births, of which 321 are explained by the fact that only one woman per household is selected for this module in the DHS. For these reasons, some models had smaller sample sizes. The relationship autonomy dimension was built from seven component variables and 18 women had missing values in any, of which 15 had only one missing value. For these 18 women, the average of the other components was filled in for the missing one(s). Various confounders also had missing values, leading to slightly smaller sample sizes in the adjusted models. As the number of missing values overall was very small (<1% in crude models and 1–7% in adjusted models) and unlikely to have influenced the results, we did not use formal methods for incorporating subjects with partially observed data.

Ethical approval for the secondary data analysis was granted by the London School of Hygiene & Tropical Medicine ethics committee on 03 July 2007 (application number 5172).

## Results

Overall, 988 of 3190 births (31%) to rural married women were delivered in a health facility. Autonomy scores showed a wide spread in the population. Relationship autonomy, for instance, ranged from 4 to the maximum 37 points for raw scores, between 14 and 27 points for cluster averages and between −18 and +17.5 for the individual deviances. The health care seeking dimension ranged from 0 to the maximum 10 points for raw scores, with few scoring low, at the cluster level the range was from 5.2 to 9.3 and the individual deviances ranged from −8 to + 4.8. (Appendix B).

In the multilevel models, adjusting for confounders and distinguishing the cluster level from the individual level, we found no effect of financial autonomy, freedom of movement or violence experience on place of delivery on either the individual or cluster level. Higher relationship autonomy showed a strong positive association with facility delivery at the cluster level (OR 1.27, 95%CI 1.14–1.43, p < 0.001) but not at the individual level (OR 1.02, 95%CI 0.99–1.05, p = 0.17). Similarly, higher autonomy in decision-making on health care seeking was strongly associated with facility delivery at the cluster level (OR 1.57, 95%CI 1.17–2.12, p = 0.001) but not the individual level (OR 1.06, 95%CI 0.99–1.13, p = 0.11) (Table 1).

**Figure 1:**
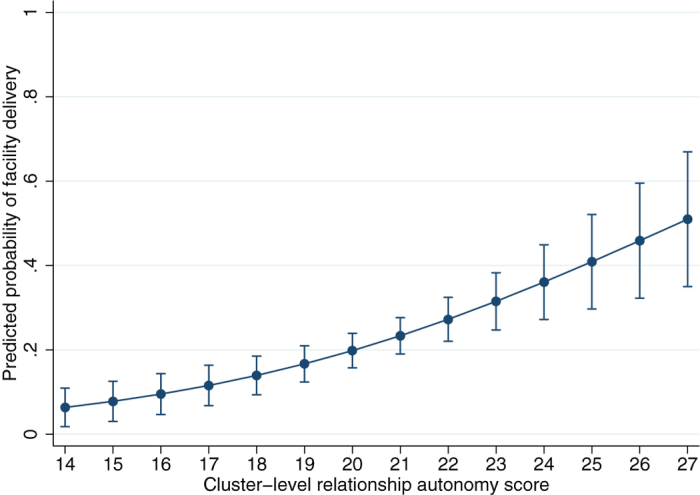
Probability of facility delivery by cluster-level relationship autonomy. This graph shows predicted probabilities (with 95% confidence intervals) from an adjusted model (n = 3116) for different relationship autonomy scores at the cluster level. The probabilities of facility delivery ranged from 6% for women from villages with low average scores (14) for relationship autonomy, to 51% of births in a facility for women from villages scoring high (27).

**Figure 2:**
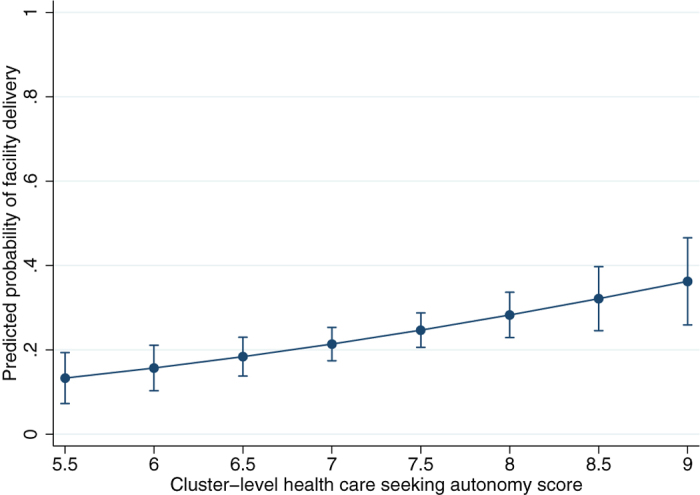
Probability of facility delivery by cluster-level health care seeking autonomy. This graph shows predicted probabilities (with 95% confidence intervals) from an adjusted model (n = 3116) for different scores of health care seeking autonomy at the cluster level. The probabilities of facility delivery ranged from 13% for women from villages with low average scores (5.5) for health care seeking autonomy, to 36% for women from villages scoring high (9).

The probability of a typical married woman in rural Zambia delivering in a health facility can be calculated as 6% if her village had the lowest score (14) for relationship autonomy, and as high as 51% if her village had the highest score (27), adjusted for the distribution of confounders including geographic access to services (Fig. 1). The probability of facility delivery for women in clusters scoring lowest (5.5) on health care autonomy was predicted as 13%, as compared to 36% in highest-scoring clusters (9 points), also adjusted for confounders (Fig. 2).

## Discussion

This analysis deepens our understanding of female autonomy in relation to care seeking for childbirth in rural Zambia, and the dimensions and levels at which autonomy operates. We found that women’s autonomy in their relationship and concerning their health care seeking are important determinants of facility delivery in this setting. Using a multilevel model with separate terms for cluster-level average autonomy and individual deviance from that average, we could discern that these factors seem to act at the community level, rather than the individual level, as may be assumed from an analysis of individual-level data. These findings reinforce an understanding that autonomy is not only multi-dimensional but also multi-layered, and as such use of the term in its broadest sense masks critical distinctions.

While gender theorists have emphasized that female autonomy should be considered across multiple levels^7^, to our knowledge, few studies have quantitatively drawn a distinction between the individual versus community level when considering the role of autonomy on a health-related outcome^21^. Acknowledging this, Matthews and Gubhaju^24^ included both individual autonomy variables and a district-level gender empowerment measure (GEM) in their study on care seeking during the antenatal period in Nepal and found that women from districts with higher GEM scores were more likely to use antenatal care.

Our findings underscore the importance of broader societal norms, which create and reinforce systems that can circumscribe or strengthen individual female autonomy. Women may have power or autonomy in their homes, but their movement outside the house may be restricted based on social norms or place of residence^21^,^25^. This finding, while somewhat novel in the maternal health field, is reflected in several veins of literature, including research on discrimination due to sex or race whereby “people and institutions engaging in discrimination adversely restrict, by judgment and action, the lives of those against whom they discriminate”^26^. Similar to the manner in which racism, as a social construct, constricts individual lives, women living within autonomy-restrictive settings may possess autonomy-promotive attitudes at the individual level, but are nevertheless embedded within, and therefore subjected to, the values of their broader group^26^.

While this is a relatively novel approach within the maternal health literature, other fields of study long ago recognized that autonomy resides within both the individual and within their broader group. In business and management literature, for example, researchers have examined the relationship between productivity (or performance) and levels of team versus individual autonomy, and found that autonomy at the individual level “may conflict with autonomy at the group level, producing countervailing influence on the cohesiveness and, indirectly, effectiveness of the work group”^27^. In practical terms, this research states that organizations or overseeing bodies aiming to empower individuals must gauge the “location of autonomy” in order to understand whether efforts toward empowerment (via reinforcement of individual autonomy, for example) would complement or run counter to the nature of the broader group^27^.

As applied to the spheres of development and public health, this suggests that studies on women’s autonomy and empowerment need to consider context, remain attentive to “normatively-governed gender stratification” systems, and capture data related to “the rights, obligations and resources granted to females versus males under different gender systems rather than on the characteristics of individual women”^21^. Public health interventions and in particular behavior change programs are currently often developed to target individuals, while changing community norms and broader conditions restricting individual choice may provide a more promising approach.

Similar to most studies on autonomy and maternal health, this study relied on household survey data collected at the individual level, which is limited in its ability to capture information regarding “social norms, social networks, individual integration into social networks, availability of social support, community-level attitudes toward health behaviors, or decision-making patterns within extended families”^15^. The surveys also rely on face validity, which has been highlighted as problematic in the autonomy literature as definitions are subject to uneven interpretation^4^. Our choice of variables was guided by data availability, rather than by a theoretical framework. This study was strengthened by the inclusion of variables related to quality and accessibility of delivery care; by adjusting for these variables we could be confident that cluster-level behaviors were not a byproduct of absent or present health services.

Our focus was on illustrating the importance of conceptualizing autonomy in several dimensions, and at the community and individual level separately, rather than on the specific findings in this particular setting. Zambia was chosen as it provided a suitable example given the availability of national data not only from the DHS, but also from a Health Facility Census, which allowed us to control for distance to care and quality of delivery care, potentially important cluster-level confounders.

Future analyses could apply a similar approach to other DHS countries, with the limitation that data on distance and quality of care will not usually be available. It is also possible to calculate population-attributable fractions for autonomy. In Zambia, we have estimated that 15% of home births could be avoided if all women lived in clusters scoring high on relationship autonomy^22^.

## Conclusions

Female autonomy is a multifaceted concept, and it is important to consider not only the dimensions of autonomy but also the levels at which autonomy operates. Power relations and gender norms at the community level may override an individual’s autonomous decisions – including preferences or behaviors related to care seeking for childbirth.

## Additional Information

**How to cite this article**: Gabrysch, S. *et al*. Autonomy dimensions and care seeking for delivery in Zambia; the prevailing importance of cluster-level measurement. *Sci. Rep.* **6**, 22578; doi: 10.1038/srep22578 (2016).

## Supplementary Material

Supplementary Information

## Figures and Tables

**Table 1:** Association of autonomy dimensions at cluster level and individual level with facility delivery.

Autonomy dimension	Crude OR* (95% CI)	p-value	Adjusted OR** (95% CI)	p-value
**Financial**	**(n = 3181)**		**(n = 2972)**	
Cluster-level	1.18 (0.92–1.52)	0.18	0.92 (0.72–1.18)	0.53
Individual-level (deviance)	1.02 (0.96–1.08)	0.60	1.00 (0.93–1.07)	0.93
**Movement**	**(n = 3186)**		**(n = 2985)**	
Cluster-level	1.26 (0.92–1.73)	0.14	1.05 (0.76–1.44)	0.78
Individual-level (deviance)	0.96 (0.88–1.04)	0.29	0.95 (0.87–1.04)	0.25
**Health care**	**(n = 3186)**		**(n = 3116)**	
Cluster-level	1.72 (1.24–1.38)	0.001	1.57 (1.17–2.12)	0.001
Individual-level (deviance)	1.04 (0.97–1.11)	0.31	1.06 (0.99–1.13)	0.11
**Relationship**	**(n = 3190)**		**(n = 3116)**	
Cluster-level	1.35 (1.21–1.51)	<0.001	1.27 (1.14–1.43)	<0.001
Individual-level (deviance)	1.04 (1.01–1.07)	0.006	1.02 (0.99–1.05)	0.17
**Violence**	**(n = 2857)**		**(n = 2841)**	
Cluster-level	0.90 (0.77–1.05)	0.17	0.90 (0.79–1.03)	0.13
Individual-level (deviance)	1.03 (0.99–1.06)	0.12	1.03 (1.00–1.07)	0.06

^*^From three-level random effects logistic regression model (levels: birth, mother, cluster).

^**^Adjusted for age, parity, education, occupation, ethnic group, religion, household wealth, husband’s education and occupation, distance to care, quality of care and other individual and cluster-level variables (see Methods for details).
