# Supplementary material for: Autonomy dimensions and care seeking for delivery in Zambia; the prevailing importance of cluster-level measurement
Source: Sci Rep. 2016 Mar 2;6:22578. doi: 10.1038/srep22578 (PMC4773858; doi:10.1038/srep22578)
Supplement: Supplementary Information [file srep22578-s1.doc]

# Supplementary information

Manuscript title

Autonomy dimensions and care seeking for delivery in Zambia; the prevailing importance of cluster-level measurement

## Authors

Sabine Gabrysch^1*^, Shannon A. McMahon^1^, Katja Siling^1,2^, Michael G. Kenward^2^, Oona M. R. Campbell^2^

## Author affiliations

^1^ Ruprecht-Karls-Universität Heidelberg, Institute of Public Health

^2^ London School of Hygiene & Tropical Medicine, Faculty of Epidemiology and Population Health

## *Corresponding author

Sabine Gabrysch

Institute of Public Health

Im Neuenheimer Feld 324

69120 Heidelberg

Germany

Tel.: +49 6221 56 5086

Email: sabine.gabrysch@uni-heidelberg.de

## Supplementary information

**Appendix A**

**Table A.1: Construction of raw scores of autonomy dimensions from component variables in the Zambia DHS 2007**

| **Autonomy dimension** | **Component variables** | **Score** | **Categories** | **Max. sum** |
| --- | --- | --- | --- | --- |
| **Financial** | Decision-making on large purchases | 5  3  0 | Woman alone  Jointly with other person  Somebody else | 10 |
|  | Decision-making on use of woman’s own earnings | 5  3  0 | Woman alone  Jointly with other person  Somebody else / no own money |  |
| **Movement** | Decision-making to visit friends and relatives | 5  3  0 | Woman alone  Jointly with other person  Somebody else | 5 |
| **Health care** | Decision-making on own health care | 5  3  0 | Woman alone  Jointly with other person  Somebody else | 10 |
|  | Getting permission for seeking medical help for oneself when sick | 5  0 | No big problem  Big problem |  |
| **Relationship** | Age at marriage | 6  5  4  3  2  1  0 | 20+ years  19-20 years  18 years  17 years  16 years  15 years  14 years or younger | 37 |
|  | Age at first birth | 6  5  4  3  2  1  0 | 22+ years  20-21 years  19 years  18 years  17 years  16 years  15 years or younger |  |
|  | Age difference to husband | 5  4  3  1  0 | Younger / same age  1-3 years older  4-6 years older  7-10 years older  10+ years older |  |
|  | Marriage type | 5  0 | Monogamous  Polygamous |  |
|  | Negotiation with partner possible on having sex and on condom use | 5  3  0 | Both possible  One possible  None possible |  |
|  | Attitudes towards wife-beating: justified for reason 1-5:   1. goes out without telling him 2. neglects children 3. argues with him 4. refuses to have sex with him 5. burns the food | 5  4  3  2  1  0 | No reason justified  1 reason justified  2 reasons justified  3 reasons justified  4 reasons justified  All 5 reasons justified |  |
|  | Attitudes towards refusal of sex: justified for reason 1-3:   1. husband has STD 2. husband has other woman 3. tired / not in mood | 5  3  1  0 | All 3 reasons justified  2 reasons justified  1 reason justified  No reason justified |  |
| **Violence experience** | Husband control behaviors: 1-6   1. Jealous/angry if talking to men 2. Frequently accuses her of being unfaithful 3. Does not permit her to meet her female friends 4. Limits her contact with her family 5. Insists on knowing where she is at all times 6. Does not trust her with any money | 6  5  4  3  2  1  0 | No control behavior  1 control behavior  2 control behaviors  3 control behaviors  4 control behaviors  5 control behaviors  All 6 control behaviors | 26 |
|  | Emotional violence by husband:  Humiliated her in front of others, threatened with hurt or harm,  insulted or made her feel bad | 5  0 | No such behaviors  Any such behaviors |  |
|  | Less severe violence by husband:   1. Slapped 2. Twisted her arm or pulled her hair 3. Pushed, shook or threw something 4. Punched with fist or harmful item 5. Kicked, dragged or beat her up | 5  4  3  2  1  0 | No such behaviors  1 behavior  2 behaviors  3 behaviors  4 behaviors  All 5 behaviors |  |
|  | Severe violence by husband   1. Tried to choke or burn her 2. Threatened / attacked with weapon 3. Hit her during pregnancy | 3  2  1  0 | No behaviors  1 behavior  2 behaviors  3 behaviors |  |
|  | 4.__Physical results of husband’s violence: e.g. cuts, bruises, injuries, deep wounds, broken bones or burns | 2  0 | No physical results  Any physical results |  |
|  | Sexual violence by husband:  Physically forced sex when not wanted, forced any sexual acts not wanted | 5  0 | No sexual violence  Any sexual violence |  |

**Appendix B**

**Table B.1: Raw autonomy score distribution**

| **Autonomy dimensions**  **Raw scores** | **% per category** |
| --- | --- |
|  |  |
| **Financial** | **n=3191** |
| Very low (0 points) | 42.0 |
| Low (3 points) | 31.0 |
| Medium (5-6 points) | 20.3 |
| High (8-10 points) | 6.7 |
|  |  |
| **Movement** | **n=3196** |
| Low (0 points) | 36.1 |
| Medium (3 points) | 42.5 |
| High (5 points) | 21.3 |
|  |  |
| **Health care** | **n=3196** |
| Very low (0-3 points) | 3.4 |
| Low (5 points) | 36.8 |
| Medium (8 points) | 33.0 |
| High (10 points) | 26.9 |
|  |  |
| **Relationship** | **n=3200** |
| Very low (4-15 points) | 15.7 |
| Low (16-22 points) | 37.1 |
| Medium (23-27 points) | 30.3 |
| High (28-37 points) | 16.9 |
|  |  |
| **Violence experience** | **n=2867** |
| High (0-16 points) | 15.4 |
| Medium (17-22 points) | 30.6 |
| Low (23-25 points) | 37.6 |
| None (26 points) | 16.5 |

**Table B.2: Cluster-level and individual-level autonomy score distribution**

| **Autonomy dimension** | **Cluster-level averages** | **Individual-level deviances** |
| --- | --- | --- |
|  |  |  |
| **Financial** | **n=203** | **n=3191** |
| Mean (SD) | 2.77 (1.12) | -0.06 (2.44) |
| Median | 2.67 | -0.33 |
| Interquartile range | 1.95 to 3.61 | -2.00 to 1.58 |
| Range | 0.18 to 5.63 | -5.63 to 8.00 |
|  |  |  |
| **Movement** | **n=203** | **n=3196** |
| Mean (SD) | 2.45 (0.88) | -0.003 (1.71) |
| Median | 2.56 | 0.10 |
| Interquartile range | 1.81 to 3.11 | -1.29 to 1.27 |
| Range | 0.35 to 4.45 | -3.9 to 4.3 |
|  |  |  |
| **Health care** | **n=203** | **n=3196** |
| Mean (SD) | 7.31 (0.85) | -0.01 (2.10) |
| Median | 7.30 | 0.24 |
| Interquartile range | 6.73 to 7.88 | -1.69 to 1.67 |
| Range | 5.21 to 9.33 | -7.97 to 4.79 |
|  |  |  |
| **Relationship** | **n=203** | **n=3200** |
| Mean (SD) | 20.56 (2.50) | 1.30 (5.34) |
| Median | 20.57 | 1.61 |
| Interquartile range | 18.75 to 22.29 | -2.24 to 5.14 |
| Range | 14.00 to 27.00 | -18.14 to 17.54 |
|  |  |  |
| **Violence experience** | **n=203** | **n=2867** |
| Mean (SD) | 20.66 (1.94) | 0.28 (4.72) |
| Median | 20.90 | 1.60 |
| Interquartile range | 19.33 to 22.07 | -2.07 to 3.50 |
| Range | 12.80 to 25.50 | -20.53 to 10.75 |
